# Increased levels of anti-PfCSP antibodies in post-pubertal females versus males immunized with PfSPZ Vaccine does not translate into increased protective efficacy

**DOI:** 10.3389/fimmu.2022.1006716

**Published:** 2022-10-25

**Authors:** Natasha KC, L. W. Preston Church, Pouria Riyahi, Sumana Chakravarty, Robert A. Seder, Judith E. Epstein, Kirsten E. Lyke, Benjamin Mordmüller, Peter G. Kremsner, Mahamadou S. Sissoko, Sara Healy, Patrick E. Duffy, Said A. Jongo, Vicente Urbano Nsue Ndong Nchama, Salim Abdulla, Maxmillian Mpina, Sodiomon B. Sirima, Matthew B. Laurens, Laura C. Steinhardt, Martina Oneko, MingLin Li, Tooba Murshedkar, Peter F. Billingsley, B. Kim Lee Sim, Thomas L. Richie, Stephen L. Hoffman

**Affiliations:** ^1^ Sanaria Inc., Rockville, MD, United States; ^2^ Vaccine Research Center, National Institute of Heath, Bethesda, MD, United States; ^3^ Naval Medical Research Center (NMRC), Silver Spring, MD, United States; ^4^ Center for Vaccine Development and Global Health, University of Maryland School of Medicine, Baltimore, MD, United States; ^5^ Institut für Tropenmedizin, Eberhard Karls Universität Tübingen and German Center for Infection Research, Tübingen, Germany; ^6^ Department of Medical Microbiology, Radboud University Medical Center, Nijmegen, Netherlands; ^7^ Centre de Recherches Medicales de Lambaréné, Lambaréné, Gabon; ^8^ Malaria Research and Training Center (MRTC), Mali National Institute of Allergy and Infectious Diseases International Centers for Excellence in Research, University of Science, Techniques and Technologies of Bamako, Bamako, Mali; ^9^ Laboratory of Malaria Immunology and Parasitology, National Institutes of Allergy and Infectious Diseases, National Institutes of Health (LMIV/NIAID/NIH), Rockville, MD, United States; ^10^ Bagamoyo Research and Training Centre, Ifakara Health Institute, Bagamoyo, Tanzania; ^11^ Ministry of Health and Social Welfare, Malabo, Equatorial Guinea; ^12^ Swiss Tropical Public Health Institute, Basel, Switzerland; ^13^ University of Basel, Basel, Switzerland; ^14^ Groupe de Recherche Action en Santé, Ouagadougou, Burkina Faso; ^15^ Centre National de Recherche et de Formation sur le Paludisme, Ouagadougou, Burkina Faso; ^16^ Malaria Branch, Division of Parasitic Diseases and Malaria, Center for Global Health, Centers for Disease Control and Prevention, Atlanta, GA, United States; ^17^ Kenya Medical Research Institute, Centre for Global Health Research, Kisumu, Kenya

**Keywords:** PfSPZ Vaccine, malaria vaccine, *Plasmodium falciparum*, PfCSP, antibodies, humoral immunity, sex, gender

## Abstract

**Background:**

While prior research has shown differences in the risk of malaria infection and sickness between males and females, little is known about sex differences in vaccine-induced immunity to malaria. Identifying such differences could elucidate important aspects of malaria biology and facilitate development of improved approaches to malaria vaccination.

**Methods:**

Using a standardized enzyme-linked immunosorbent assay, IgG antibodies to the major surface protein on *Plasmodium falciparum* (Pf) sporozoites (SPZ), the Pf circumsporozoite protein (PfCSP), were measured before and two weeks after administration of a PfSPZ-based malaria vaccine (PfSPZ Vaccine) to 5-month to 61-year-olds in 11 clinical trials in Germany, the US and five countries in Africa, to determine if there were differences in vaccine elicited antibody response between males and females and if these differences were associated with differential protection against naturally transmitted Pf malaria (Africa) or controlled human malaria infection (Germany, the US and Africa).

**Results:**

Females ≥ 11 years of age made significantly higher levels of antibodies to PfCSP than did males in most trials, while there was no indication of such differences in infants or children. Although adult females had higher levels of antibodies, there was no evidence of improved protection compared to males. In 2 of the 7 trials with sufficient data, protected males had significantly higher levels of antibodies than unprotected males, and in 3 other trials protected females had higher levels of antibodies than did unprotected females.

**Conclusion:**

Immunization with PfSPZ Vaccine induced higher levels of antibodies in post-pubertal females but showed equivalent protection in males and females. We conclude that the increased antibody levels in post-pubertal females did not contribute substantially to improved protection. We hypothesize that while antibodies to PfCSP (and PfSPZ) may potentially contribute directly to protection, they primarily correlate with other, potentially protective immune mechanisms, such as antibody dependent and antibody independent cellular responses in the liver.

## Introduction

In 2020, malaria caused 241 million clinical episodes and 627,000 deaths (1), the highest number of deaths since 2012. The worsening situation has occurred despite an annual investment of >$3 billion in intensive control measures, indicating a saturation of capacity to achieve further impact (2, 3). The WHO estimated that there were more deaths in Africa from malaria than from COVID-19 in 2020 (4), resulting in 40-fold more disability life years (DALYS) lost from malaria in Africa in 2020 than from COVID-19 from February 2020 to March 2021 (5).

Despite the global malaria control efforts, progress has slowed in recent years and there is an urgent need for highly effective malaria vaccines. A malaria vaccine, RTS,S/AS01, has been recently recommended for implementation in young African children by the World Health Organization based on the results of a pilot implementation program in Ghana, Malawi, and Kenya in 920,000 infants in which the vaccine reduced malaria hospitalizations by 21% and severe malaria by 30% (6). Our long-term goal is the development of a much more effective malaria vaccine that can be used to eliminate malaria because it prevents infection with Pf. We use whole *Plasmodium falciparum* (Pf) sporozoites (SPZ), the entire parasite, as the immunogen in our vaccines (7).

Our first-generation malaria vaccine is Sanaria^®^ PfSPZ Vaccine, which is made up of radiation-attenuated, aseptic, purified, cryopreserved PfSPZ. It has been tested in 21 clinical trials in the United States (US), Europe, and six African countries (8–29). A meta-analysis of 13 double-blind, placebo-controlled trials of PfSPZ Vaccine, 11 of which were conducted in Africa, revealed no significant difference in adverse event patterns between vaccinees and controls who received normal saline (NS) (16–21, 25–29). Vaccine efficacy (VE) reached 100 percent against homologous (same Pf strain as the vaccine, NF54) controlled human malaria infection (CHMI) at 3-7 weeks after the last dose of vaccine (17, 27, 30), and 78 percent against heterologous (Pf7G8 strain) CHMI at 3 and 9-10 weeks (14, 28), and lasted for at least 14 months against homologous (13) and 8 months against heterologous CHMI (15). VE against Pf infection has been demonstrated in field trials in African adults to last at least 18 months and vary from 47 to 85 percent depending on the trial, dosage regimen and population assessed (29). This protection is seen despite antibody and cellular immune responses that are many-fold lower than in malaria-naive adults in Germany or the US.

Vaccination-induced protective immunity is mediated by a complex combination of innate, humoral, and cell-mediated immune responses (31–36). The influence of biological sex on immunity has gathered attention in recent years, and a growing body of data suggests that sex-specific effects may result in variable immunological and efficacy outcomes after vaccination (32). Females tend to have greater antibody responses than males, higher basal immunoglobulin levels and higher B cell numbers (32, 33, 35–37).

In all our clinical trials we have assessed, in the same laboratory, the IgG antibody responses to the major protein on the surface of PfSPZ, the Pf circumsporozoite protein (CSP), prior to immunization and 2 weeks after the last immunizing dose. In a number of the trials, especially the field trials, anti-PfCSP antibody levels were higher in vaccinees who were protected as compared to those who were not protected (16, 25, 27). In this paper we report our analysis of the comparative anti-PfCSP antibody responses and protective efficacy between male and female vaccinees in 11 clinical trials in the US, Germany, Kenya, Tanzania, Mali, Burkina Faso, and Equatorial Guinea.

## Methods

### Selection of clinical trials

All clinical trials of PfSPZ Vaccine were considered for inclusion. Trials were included if they met the following criteria: 1) PfSPZ Vaccine was administered by direct venous inoculation (DVI); 2) The trial included female participants; 3) Datasets including participant demographics, net OD 1.0 (see ELISA methods for definition of Net OD 1.0) anti-PfCSP levels by ELISA and vaccine efficacy outcomes (when assessed) were available for analysis. Because participants were not assessed for biological sex, the data collected on sex are represented by self-identified or parent-identified gender. To assess differences in potential effects of changes in the hormonal milieu associated with puberty, the data were divided into study participants < 11 years of age and ≥ 11 years of age as part of the analysis.

### IgG antibodies to PfCSP by ELISA

IgG antibodies to the Pf circumsporozoite protein (CSP) were assessed by ELISA as previously described (38). Briefly, 96-well plates (Nunc MaxiSorp Immuno Plate) were coated overnight at 4°C with 2 µg/mL of a nearly full length recombinant PfCSP protein [described in (38)] in 50 µL per well in coating buffer (Coating Solution Concentrate Kit, KPL, Catalog# 5150-0014). Plates were washed three times with 2 mM imidazole, 160 mM NaCl, 0.02% Tween 20, 0.5 mM EDTA and blocked with 1% Bovine Serum Albumin (BSA) blocking buffer (10% BSA Diluent/Blocking Solution, KPL, Catalog# 5140-0006) containing 1% non-fat dry Milk for 1 h at 37°C. Plates were washed three times and serially diluted serum samples (in triplicates) were added and incubated at 37°C for 1 h. After three washes, peroxidase labelled goat anti-human IgG (Anti-Human IgG (H+L) Antibody, Peroxidase-Labeled, KPL, Catalog #5220-0330) was added at a dilution of 0.1 µg/ml and incubated at 37°C for 1 h. Plates were washed three times, ABTS peroxidase substrate was added for plate development, and the plates were incubated for 75 min at room temperature. The plates were read with a Spectramax Plus 384 microplate reader (Molecular Devices) at 405 nm. The data were collected using SoftMax Pro GXP v5 and fit to a 4-parameter logistic curve, to calculate the serum dilution yielding an optical density reading of 1.0 (OD 1.0). A negative control (pooled sera from non-immune individuals from a malaria free area) was included in all assays. Serum from an individual with anti-PfCSP antibodies was used as a positive control. The same negative and positive controls were used in all assays. The assay was conducted on sera obtained prior to immunization and 2 weeks after the last immunization. Samples were considered positive if the difference between the post-immunization OD 1.0 and the pre-immunization OD 1.0 (net OD 1.0) was ≥50 and the ratio of the post-immunization OD 1.0 to pre-immunization OD 1.0 (ratio) was ≥3.0.

### Statistical and meta-analysis methods

The Net OD 1.0 ELISA anti-PfCSP levels were calculated for each participant in a trial to compare the antibody levels between female and male participants. First, the net OD 1.0 ELISA anti-PfCSP levels were obtained by calculating the difference between pre-immunization and two weeks post last immunization levels measured for each participant. Then, the negative net antibodies were replaced with a value of 1 for the logarithmic presentation of data. Finally, the net antibody levels between female and male participants were compared using the Kruskal-Wallis test (SAS 9.4). The non-parametric Wilcoxon-Mann-Whitney test was used to determine statistical significance for fold change values of antibody levels.

The protection risk ratio (RR) between male and female vaccine participants was compared to evaluate the vaccine efficacy in males and females. The RR as a parameter does not depend on aspects of study design, which vary between studies. This feature supported comparing multiple clinical trial outcomes obtained from different populations, population sizes, and vaccine doses. The RR was obtained from (39).


(1) RR=an1cn2

Where *a* was the number of protected male participants in a trial vaccine group, *n*_1_ was the total number of males in that trial’s vaccine group. The *c* and *n*_2_ were the number of protected females and total female participants in the vaccine group. In a random-effects meta-analysis, *Ln*(*RR*) was each trial’s study effect (*r_i_*). In the random-effects meta-analysis, the 95% confidence interval (CI) for RR was calculated using (2):


(2) $$CI={\hat{r}}_{i}\pm 1.96\times \hat{\sigma }$$

Where σ for was the each trial estimated standard error obtained from (3):


(3) σ^i2=1a−1n1+1c−1n2

The z-statistic value for each trial was then estimated by (4):


(4) zi=r^iσ^i

We obtain the two-tailed p-value for a trial by *p*=2[ 1−*Φ*(*z*) ], where *Φ*(*z*) was the standard normal cumulative distribution (39). Lastly, the overall RR of all trials was calculated using the random-effects modified inverse variance method for trial weights. The modified weight was calculated by (5):


(5) $$w_{i}={\tilde{w}}_{inv}+\ln(n_{1}+n_{2})$$

The inverse variance estimates study weight and can be presented by w˜inv=1σ^i . The logarithmic summation modifies the study weights (modified variance) to overcome the possible small study size problem due to numerous small sample size trials (40, 41). Finally, the *Q*-statistics and *I^2^* values for the random-effect analysis were measured to report the heterogeneity of the meta-analysis on male and female vaccine efficacy.

## Results

### Clinical trials

Data from 11 clinical trials were available for analysis (**Table 1**). These included 8 adult (age ≥ 18 years) trials with immunology and efficacy data; 1 trial with infants (ages 5 to 12 months) and children (ages 1 to 9 years) with immunology for all ages and efficacy for the infant cohort; and 2 trials with infants (ages 6-12 months), children (ages 1-17 years) and adults (age ≥ 18 years) with immunology data for all 3 age groups but efficacy data only for adults. Trials conducted in the US and Germany enrolled malaria naïve adults; efficacy was assessed using controlled human malaria infection (CHMI). Trials conducted in sub-Saharan Africa (Tanzania, Equatorial Guinea, Burkina Faso, Kenya, Mali) enrolled participants with varying degrees of prior exposure to Pf; efficacy, when evaluated, was assessed against either naturally acquired infection (Kenya, Mali, Burkina Faso) or CHMI (Tanzania, Equatorial Guinea).

**Table 1:** Characteristics of the individual trials included in this analysis.

Study (Country, year trial started)	Vaccinees Male/Female	Dose	Dosing Interval (days)	Efficacy Assessment
*US and German Adults age ‗18 years*				
VRC 312 (USA, 2011) (9) *(NCT01441167)*	5/9	1.35x10^5^ PfSPZ	Group 4a – 1, 29, 113, 141 and 189 Group 4b – 1, 29, 57, 85 and 134 Group 4c – 1, 29, 57 and 106	CHMI (NF54) 3 weeks post final dose
WRAIR 2080 (USA, 2014)) (14) *(NCT02215707)*	20/14	2.7x10^5^ PfSPZ	1, 29, 57, 85 and 141	CHMI (NF54, 7G8) 3 weeks post final dose
		4.5x10^5^ PfSPZ	1, 57 and 113	
Warfighter 2 (USA, 2016) (23) *(NCT02601716)*	36/21	4.5x10^5^ PfSPZ	1, 3, 5, 7 and 113	CHMI 12 weeks post final dose
		9.0x10^5^ PfSPZ	1, 57 and 113	
		1.8x10^6^ PfSPZ	1, 57 and 113	CHMI 24 weeks post final dose
		2.7x10^6^ PfSPZ (1^st^ dose) then 9.0x10^5^ PfSPZ	1, 57 and 113	
MAVACHE (Germany, 2016) (28) *(NCT02704533)*	8/4	9.0x10^5^ PfSPZ	1, 8 and 29	CHMI 3 weeks post final dose
*African children and adults age ≥ 11 years*				
MLSPZV1 (Mali, 2014) (16) *(NCT01988636)*	35/7	2.7x10^5^ PfSPZ	1, 29, 57, 85 and 141	Naturally acquired infection 4 to 24 weeks post final dose
BSPZV2 (Tanzania, 2015) (19, 22) *(NCT02613520)*	13/12	9.0x10^5^ or 1.8x10^6^ PfSPZ	1, 57 and 113	CHMI (age ≥ 18 years) 3-11 weeks post final dose
MLSPZV2 (Mali, 2016) (27) *(NCT02627456)*	41/15	1.8x10^6^ PfSPZ	1, 57 and 113	Naturally acquired infection 0 to 24 weeks post final dose
BFSPZV1 (Burkina Faso, 2016) (29) *(NCT02663700)*	21/18	2.7x10^6^ PfSPZ	1, 57 and 113	Naturally acquired infection 0 to 24 weeks post final dose
EGSPZV2 (Equatorial Guinea, 2016) [ (24), Jongo et al., manuscript in preparation] *(NCT02859350)*	23/4	2.7x10^6^ PfSPZ	1, 57 and 113	CHMI (age ≥ 18 years) 14-33 weeks post final dose
EGSPZV3 (Equatorial Guinea, 2018) [(26) Jongo et al., AJTMH 2022]	64/13	9.0x10^5^ PfSPZ	Group 1 - 1, 3, 5, 7 and 113 Group 2 - 1, 3, 5 and 7 Group 3 - 1, 3, 5, 7 and 29 Group 4 - 1, 8 and 29	CHMI 3 weeks post final dose
*African infants and children age 5 months – 11 years*				
BSPZV2 (Tanzania, 2015) (19) *(NCT02613520)*	14/20	4.5x10^5^, 9.0x10^5^ or 1.8x10^6^ PfSPZ	1, 57 and 113 days	N/A
KSPZV1 (part 1) (Kenya, 2016) (42) *(NCT02687373)*	20/25	1.35x10^5^, 2.7x10^5^ or 4.5x10^5^	1 dose	N/A
		9.0x10^5^ or 1.8x10^6^ PfSPZ	1 and 57 days	
KSPZV2 (part 2) (Kenya, 2016) (20) *(NCT02687373)*	109/88	4.5x10^5^, 9.0x10^5^ or 1.8x10^6^ PfSPZ	1, 57 and 113 days	Naturally acquired infection 2 to 52 weeks post final dose
EGSPZV2 (Equatorial Guinea, 2016) (Jongo et al., manuscript in preparation) *(NCT02859350)*	18/15	1.8x10^6^ PfSPZ	1, 57 and 113 days	N/A

### Antibodies to PfCSP by ELISA by sex

**Figure 1:**
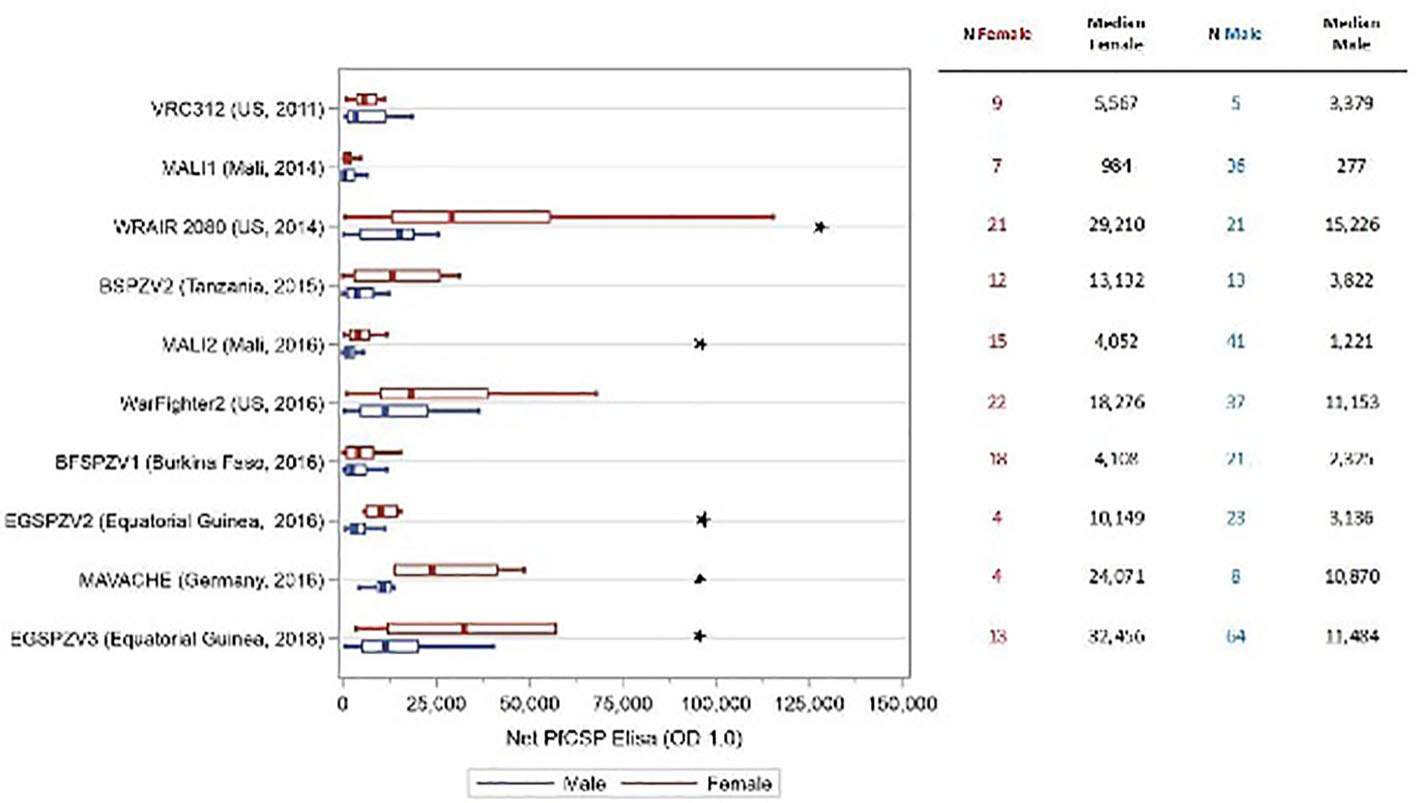
Net OD 1.0 PfCSP antibody level by sex in study participants age ≥ 11 years receiving PfSPZ Vaccine. Study name and time of study start are shown in the left of the figure, with the number of participants of each sex and the median net OD 1.0 PfCSP antibody levels for each sex in the right-hand columns. Box plots display the median, interquartile range and minimum/maximum for each trial with female participants represented in red and male participants in blue. The difference in net PfCSP antibody responses between females and males was statistically significant (p<0.05, Kruskal-Wallis test) in 5 of the trials (★).

**Figure 2:**
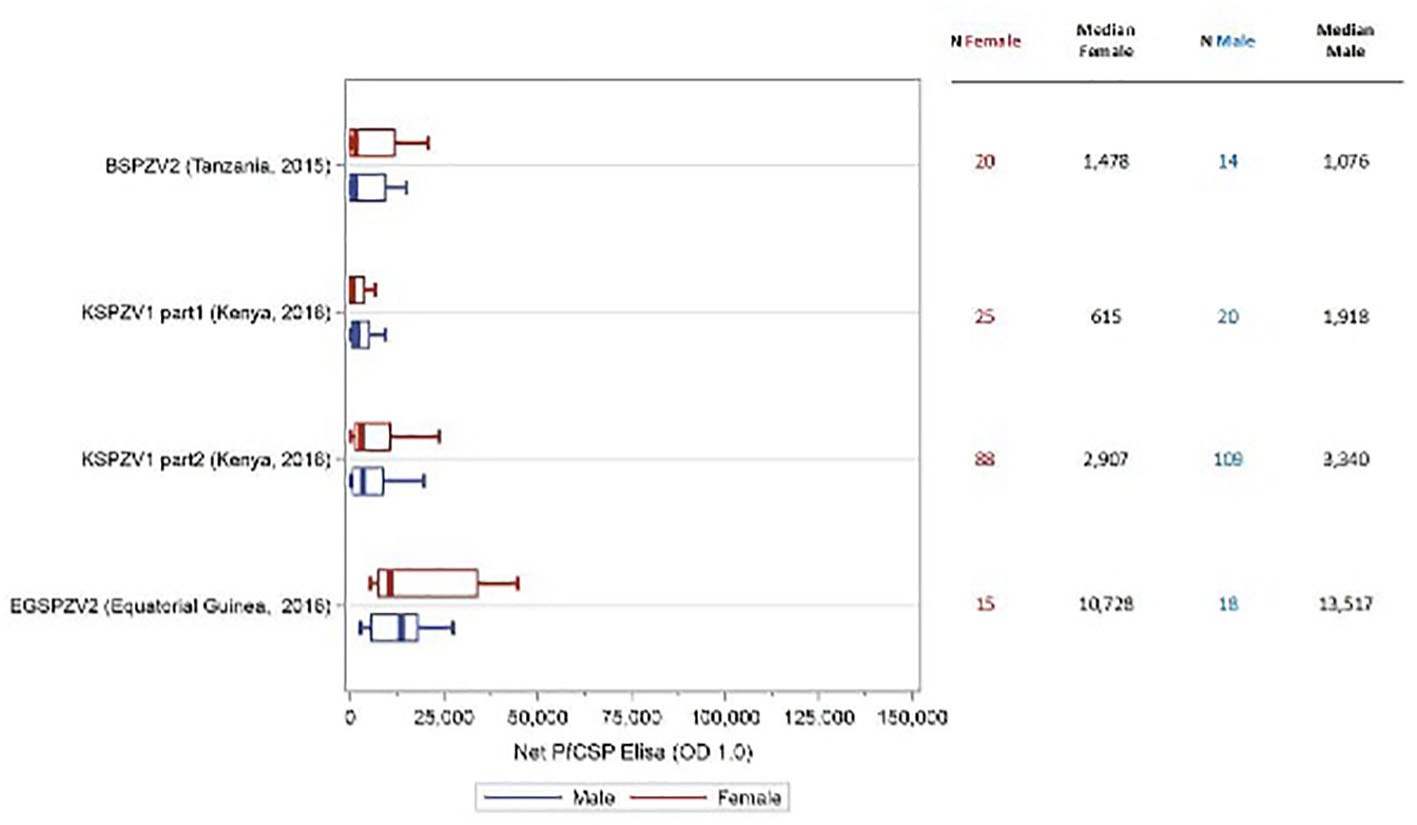
Net OD 1.0 PfCSP antibody level by sex in study participants age < 11 years receiving PfSPZ Vaccine. Study name and time of study start are shown in the left of the figure, with the number of participants of each sex and the median net PfCSP antibody levels for each sex in the right-hand columns. Box plots display the median, interquartile range and minimum/maximum for each trial with female participants represented in red and male participants in blue. There was no difference in the net PfCSP antibody responses between females and males in this age group.

In all trials, antibody levels against PfCSP were assessed prior to the first dose of vaccine and 2 weeks after the final vaccine dose. The antibody level was the serum dilution at which the optical density (OD) was 1.0. The net OD 1.0, the difference between the post- and pre-vaccination OD 1.0 levels, is reported. As reported in prior studies, antibody levels for males and females combined were substantially higher in adult study participants from sites where malaria is not endemic compared with malaria endemic areas (16, 17, 19) [Jongo, unpublished]. Antibody levels at sites located in Tanzania and Equatorial Guinea where infants to adults were assessed, correlated inversely with age (19) [Jongo, unpublished]. Net OD 1.0 PfCSP antibody levels were higher in female study participants compared with male participants in 10 of 12 trials (**Figure S1**); in 5 trials (EGSPZV2 (Equatoria Guinea, 2016), EGSPZV3 (Equatoria Guinea, 2018), MLSPZV2 (Mali 2) (Mali, 2014), WRAIR 2080 (US, 2014) and MAVACHE (Germany, 2016)) this difference was statistically significant. Net PfCSP antibody levels were higher in males in three trials – BSPZV2 (Tanzania, 2015) and KSPZV1 (Kenya, 2016). All 3 trials included children, and all participants in the Kenya trials were less than 9 years of age. When the trial data were segregated according to age ≥ or < 11 years old, all studies showed higher net PfCSP antibody levels in females age ≥ 11years compared with males, with the difference significant in 5 trials (**Figure 1**). In the 4 clinical trials with infants and children, the net PfCSP antibody levels in participants under age 11 years were not significantly different, but levels were higher in males in 3 of the 4 trials (**Figure 2**).

### Vaccine efficacy by sex

**Figure 3:**
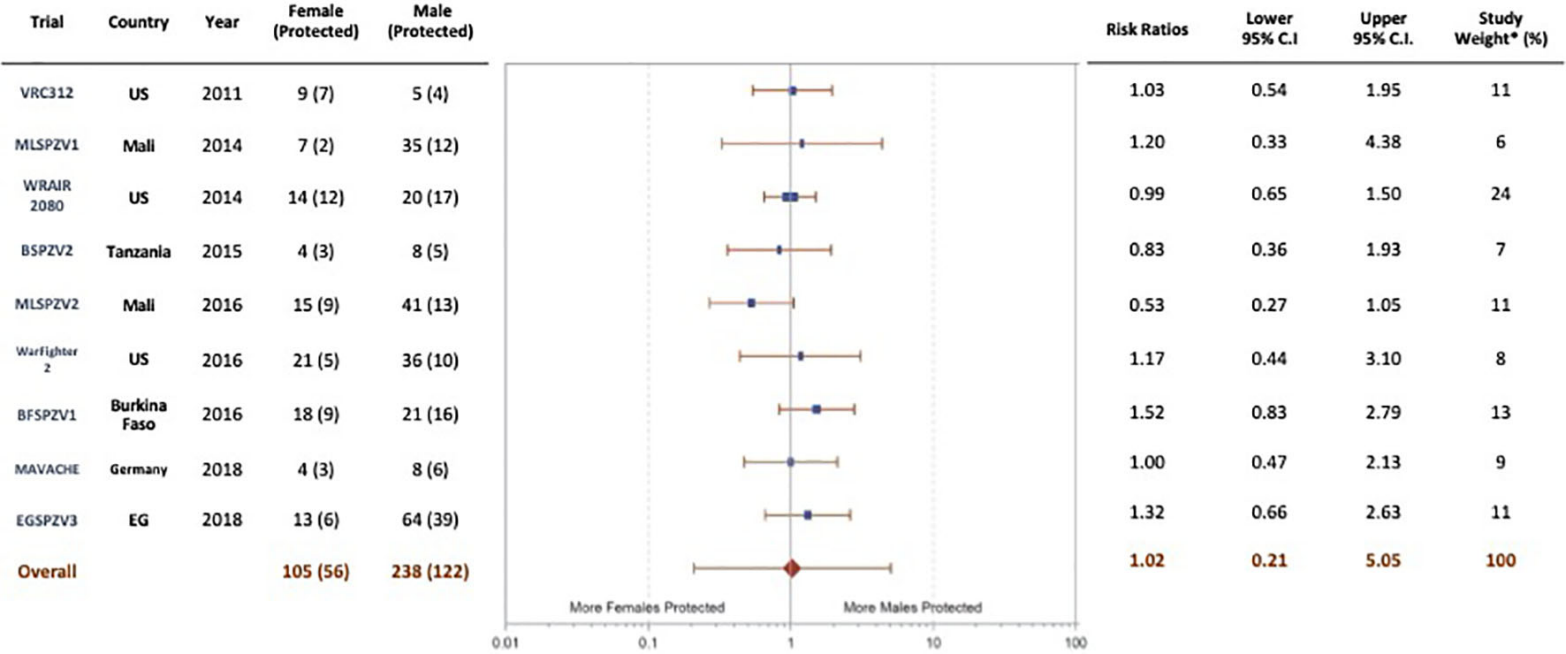
Protection status by sex in adult vaccinees (age ≥ 18 years) in trials of PfSPZ Vaccine, random-effects model. Although one trial (MLSPZV2) showed a trend towards a significant difference in vaccine efficacy favoring females, the conclusion of the meta-analysis was no difference in the efficacy of PfSPZ Vaccine in males compared with females (weighting – modified variance; *I^2^*-11.7% (-66%, 53%); p=0.97, chi-squared).

In the adult trials, vaccine efficacy was determined by CHMI at predetermined time points after the final vaccine dose or by natural exposure over a 24-week period after the final vaccine dose (**Table 1**). A meta-analysis of vaccine efficacy by sex (**Figure 3**) was done for 9 of the 10 trials in which protective efficacy was assessed in adults. The EGSPZV2 (Equatorial Guinea, 2016) had only 1 female who participated in CHMI (and was protected) and was not included in the analysis. In one trial, the MLSPZV2 (Mali 2) (Mali, 2016) trial, meta-analysis demonstrated a trend towards greater vaccine efficacy in females (RR 0.53, CI 0.28 - 1.01, p=0.057, chi-squared). However, the overall results of the meta-analysis demonstrated no difference in protective efficacy by sex (RR 1.02, CI 0.21-5.05, p=0.96, chi-squared). In the only pediatric trial to assess vaccine efficacy, KSPZV1 (part 2) (Kenya, 2016), there was no difference in efficacy by sex (RR 1.06, CI 0.76-1.39, p=0.76, chi-squared).

### Antibodies by sex and protection status

Net OD 1.0 PfCSP antibody levels by sex and protection status within each of the 7 individual trials of adults in which sample sizes were adequate to make comparisons (MLSPZV1 (Mali 1) (Mali, 2014), MLSPZV2 (Mali 2) (Mali, 2016), WRAIR 2080 (US, 2014), Warfighter 2 (US, 2016), BFSPZV1 (Burkina Faso, 2016), MAVACHE (Germany, 2016), EGSPZV3 (Equatorial Guinea, 2018)) did not yield a consistent relationship among antibody level, protection and sex (**Figure S2**). Among participants who were protected, antibody levels were higher in females than in males in 6 of 7 trials and the differences were statistically significant in 3 (WRAIR 2080 (US, 2014), MAVACHE (Germany, 2016), EGSPZV3 (Equatorial Guinea, 2018)) of the 6 trials. In 3 trials (WRAIR 2080 (US, 2014), Warfighter 2(US, 2016), and BFSPZV1 (Burkina Faso, 2016)), antibody levels were significantly higher in protected vs unprotected females. In 2 trials (MLSPZV1 (Mali 1) (Mali, 2014) and MLSPZV2 (Mali 2) (Mali, 2016)), antibody levels were significantly higher in protected vs unprotected males and in one additional trial (EGSPZV3 (Equatorial Guinea, 2018)) the difference was borderline significant (p=0.059).

## Discussion

In 100% of ten clinical trials in Mali, Tanzania, Burkina Faso, Equatorial Guinea, the US, and Germany females ≥11 years of age (**Figure 1**) made higher levels of antibodies to PfCSP than did males, and these differences were significant in five of the ten studies. In contrast, in four studies in participants <11 years old in Africa, there were no significant differences in levels of antibodies to PfCSP between females and males, and in three of the four studies, males had higher levels of antibodies (**Figure 2**). These findings are consistent with prior reports on sex differences in vaccine induced antibody responses. Adult females, for example, have shown stronger antibody responses to immunizations for influenza, hepatitis B, herpes virus, yellow fever, rabies, and smallpox virus than males (7, 31, 33, 36, 43). Sex differences in humoral immunity exist throughout life in some cases, while in others, such as appears to be the case with PfSPZ Vaccine, differences are found only after puberty, implying that genes and hormones are both likely involved (31).

Although females have shown higher vaccine-induced antibodies in many studies, it has not been consistently linked to increased vaccination effectiveness in females (44, 45). In our case, the differences in antibody responses between males and females were not mirrored by differences in efficacy; protection against CHMI or against transmission in the field appeared not to be influenced by sex (**Figure 3**). This suggests that other immune mechanisms, such as antibody-dependent or antibody-independent cell-mediated responses, are the major determinants of protection. However, we have not systematically assessed the functional capacity of antibodies in the sera of females vs. males to inhibit PfSPZ invasion of hepatocytes, which has been significantly associated with protection in some clinical trials, even when anti-PfCSP antibody level was not significantly associated (9). It is generally believed that PfSPZ-based vaccination protects against malaria infection through CD8 T cell responses that home to the liver, although other mechanisms may be involved as well (8). We surmise that antibody responses may correlate with other responses more mechanistically involved in protection, as suggested in prior publications on PfSPZ Vaccine (8, 9) and thereby act as a biomarker. This is consistent with the finding that, depending on the trial, antibody responses in non-protected individuals in one trial may be higher than antibody responses in protected individuals in another trial. For example, antibody responses in non-protected individuals in EGSPZV3 (Equatorial Guinea, 2018) (**Figure S2G**) were higher in both males and females than responses in protected individuals in MLSPZV1 (Mali 1) (Mali, 2014) (**Figure S2A**), MLSPZV2 (Mali 2) (Mali, 2016) (**Figure S2B**) or BFSPZV1 (Burkina Faso, 2016) (**Figure S2E**). If antibody levels were the primary determinants of protection, this would not be the case.

A consistent finding from our studies is that individuals with prior malaria exposure, such as African adults, have significantly lower antibody responses to PfCSP than malaria naïve adults (**Figure 1**, **Figure S1**) (14, 16, 17, 19, 23, 27). We think this is primarily due to immune dysregulation due to lifelong exposure to malaria parasites, but elimination of the PfSPZ for immunization by naturally acquired adaptive immune responses and immunosuppression due to concomitant helminth and other infections may also contribute (46). Interestingly, in trials including African infants and children there is a negative correlation between age and antibodies to PfCSP with the highest levels in infants and young children (19). Antibody levels in these children approach the responses seen with malaria-naive adults (19). Regardless, in participants ≥ 11 years of age, antibody levels were higher in females than their male counterparts despite the degree of prior malaria exposure (**Figure 1**).

An effect of dose and dosing interval was not specifically examined in this analysis. Antibody levels appear to increase with increasing total vaccine dose in groups with similar degrees of prior exposure to Pf in both males and females (**Figure 1**). Regardless of the dose and dosing interval used, all trials evaluating children and adults ≥ 11 years of age, antibody levels were higher in females compared with males (**Figure 1**).

In this study, the interplay between sex, antibody levels and protection was not straightforward. In three of seven trials with sufficient data for evaluation (one in Burkina Faso, two in the US), protected females showed statistically significantly higher antibody responses than non-protected females and males did not (**Figures S2C–E**) while in two different trials (both in Mali), protected males showed statistically significantly higher antibody responses than non-protected males and females did not (**Figures S2A, B**). In two trials (Germany and Equatorial Guinea), there were no significant differences between protected and unprotected males or females (**Figures S2F, G**). At this point, we are not able to explain these differences.

The finding that sex-related differences in protection were not revealed in this study has important practical implications. For example, there is no need to consider varying immunization regimens between males and females. Nevertheless, it will be important to continue monitoring for sex-related differences as the clinical development program for PfSPZ-based vaccines moves forward.

## Data availability statement

The raw data supporting the conclusions of this article will be made available by the authors, without undue reservation.

## Ethics statement

The study we present for publication is an analysis of data from multiple contributing studies. For every study included in this analysis: 1. Each study was individually IRB/ethics committee approved – in some cases this was one or more institutional IRB with or without a national IRB. 2. All studies required informed consent. For the studies enrolling participants under the age of 18, parental or guardian consent was a requirement for study participation. The host country definition for consent requirements was used for each trial. 3. The analysis of antibody responses and the collection of demographics, including sex, was explicitly included in each study protocol from which the data presented in this manuscript was derived. 4. The full list of ethical review committees, by trial: 1. VRC 312 - US National Institute of Allergy and Infectious Diseases (NIAID; National Institutes of Health [NIH]) IRB 2. Mali 1 - Faculté de Médecine de Pharmacie et d’OdontoStomatologie [FMPOS] IRB (Bamako, Mali); US National Institute of Allergy and Infectious Diseases (NIAID; National Institutes of Health [NIH]) IRB 3. WRAIR 2080 – Walter Reed Army Institute of Research IRB 4. BSPZV2 – Ifakara Health Institute IRB; National Institute for Medical Research (Tanzania) IRB 5. Mali 2 - Faculté de Médecine de Pharmacie et d’OdontoStomatologie [FMPOS] IRB (Bamako, Mali); US National Institute of Allergy and Infectious Diseases (NIAID; National Institutes of Health [NIH]) IRB 6. Warfighter 2 – Naval Medical Research Center IRB; University of Maryland IRB 7. BFSPZV1 – Burkina Faso Ethics Committee for Health Research (Burkina Faso); University of Maryland IRB 8. KSPZV1 – Kenya Medical Research Institute (KMRI) IRB; US Centers for Disease Control and Prevention (CDC) IRB 9. EGSPZV2 - Ifakara Health Institute IRB; Comité Ético Nacional de Guinea Ecuatorial (CENGE) 10. MAVACHE - Ethikkommission der Medizinischen Fakultat und am Universitatsklinikum Tübingen (Tübingen, Germany) 11. EGSPZV3 - Ifakara Health Institute IRB; Comité Ético Nacional de Guinea Ecuatorial (CENGE). Written informed consent to participate in this study was provided by the participants’ legal guardian/next of kin.

## Author contributions

SH, TR, LC, SC, and NK conceived, designed, and defined the analysis plan, and interpreted results. NK performed and analyzed the antibody assays. PR developed algorithms, gathered and analyzed the data. SH, TR, LC, and NK drafted the manuscript. RS, JE, KL, BM, PK, MS, SH, PD, SJ, VN, SA, SS, MLa, LS, and MO conducted the clinical trials, TM provided regulatory support, PB provided training and laboratory support, BS provided the investigational product, and TR and LC supervised the clinical trials for the sponsor. All authors read, commented on, and approved the final version of the manuscript.

## Funding

The clinical trials were funded in whole or in part with funds from the following: US National Institute of Allergy and Infectious Diseases, National Institutes of Health, including the Division of Microbiology and Infectious Diseases, the Laboratory of Malaria Immunology and Vaccinology, and the Vaccine Research Center, The Department of Defense Joint Warfighter Medical Research Program, The US Army Medical Research and Development Command, Military Infectious Diseases Research Program, The Naval Medical Research Center Advanced Medical Development Program, The Ministry of Health and Social Welfare and the Ministry of Mines and Hydrocarbons, Equatorial Guinea, Marathon EG Production Limited, Noble Energy, Atlantic Methanol Production Company, and EG Liquified Natural Gas, and German Federal Ministry of Education and Research (BMBF) through the German Center for Infection Research (DZIF). Sanaria, Inc. funded the data analysis. Marathon EG Production Limited, Noble Energy, Atlantic Methanol Production Company, and EG Liquified Natural Gas were not involved in the study design, the collection, analysis or interpretation of data, the writing of this article, or the decision to submit it for publication.

## Acknowledgments

We wish to acknowledge the invaluable support of the Sanaria Manufacturing, Quality, Regulatory and Clinical Teams and Protein Potential Assays Team, Rockville MD, USA: especially Yonas Abebe, Anusha Gunasekera, Elizabeth Saverino, Mei-Chun Chen, Abraham Eappen, Tao Li, Anita Manoj, Richard Stafford, Adam Richman, Adam Ruben, Yun Wu, Aderonke Awe, Asha Patil, LiXin Gao, Faith Beams, Virak Pich, Keith Nelson, Yingda Wen, Bing Jiang, Maria Orozco, Rui Xu, James Overby, Steve Matheny, Yeab Getachew, Enni Fomumbod, Mary King, Michelle Laskowski, Patricia De La Vega, Tint Wai, Jonathan Jackson, Meghan Marquette, Henry Huang, and Debbie Padilla. We also warmly thank the many institutions, investigators and their teams who were or are involved in the conduct of completed, ongoing and planned trials of PfSPZ products, for their collaboration.

## Conflict of interest

NK, LC, PR, SC, TM, PB, BS, TR and SH are salaried employees of Sanaria Inc., the developer and owner of PfSPZ Vaccine and sponsor of the clinical trials. In addition, BS and SH have a financial interest in Sanaria Inc.

The remaining authors declare that the research was conducted in the absence of any commercial or financial relationships that could be construed as a potential conflict of interest. The authors declare this study received funding from Sanaria Inc. 

The funder had the following involvement in the study: proposal of the study hypothesis, conduct of the ELISAs and data analysis.

## Publisher’s note

All claims expressed in this article are solely those of the authors and do not necessarily represent those of their affiliated organizations, or those of the publisher, the editors and the reviewers. Any product that may be evaluated in this article, or claim that may be made by its manufacturer, is not guaranteed or endorsed by the publisher.
