# Supplementary material for: Increased levels of anti-PfCSP antibodies in post-pubertal females versus males immunized with PfSPZ Vaccine does not translate into increased protective efficacy
Source: Front Immunol. 2022 Oct 25;13:1006716. doi: 10.3389/fimmu.2022.1006716 (PMC9641621; doi:10.3389/fimmu.2022.1006716)
Supplement: Supplementary file 1 [file DataSheet_1.pdf]

Supplementary Material

Figure S1. Net OD 1.0 PfCSP antibody level by sex in study participants receiving PfSPZ Vaccine. Study name and time of study start are shown in the left of the figure, with the number of participants of each sex and the median net OD 1.0 PfCSP antibody levels for each sex in the right-hand columns. Box plots display the median, interquartile range and minimum/maximum for each trial with female participants represented in red and male participants in blue. The difference in net OD 1.0 PfCSP antibody responses between females and males was statistically significant ($p<0.05$, Kruskal-Wallis test) in 5 of the trials (★), including three trials in Africa (Mali 2 (Mali, 2016), EGSPZV2 (Equatorial Guinea, 2016) and EGSPZV3 (Equatorial Guinea, 2018)), one in the US (WRAIR 2080 (US, 2014)) and one in Germany (MAVACHE (Germany, 2016)).

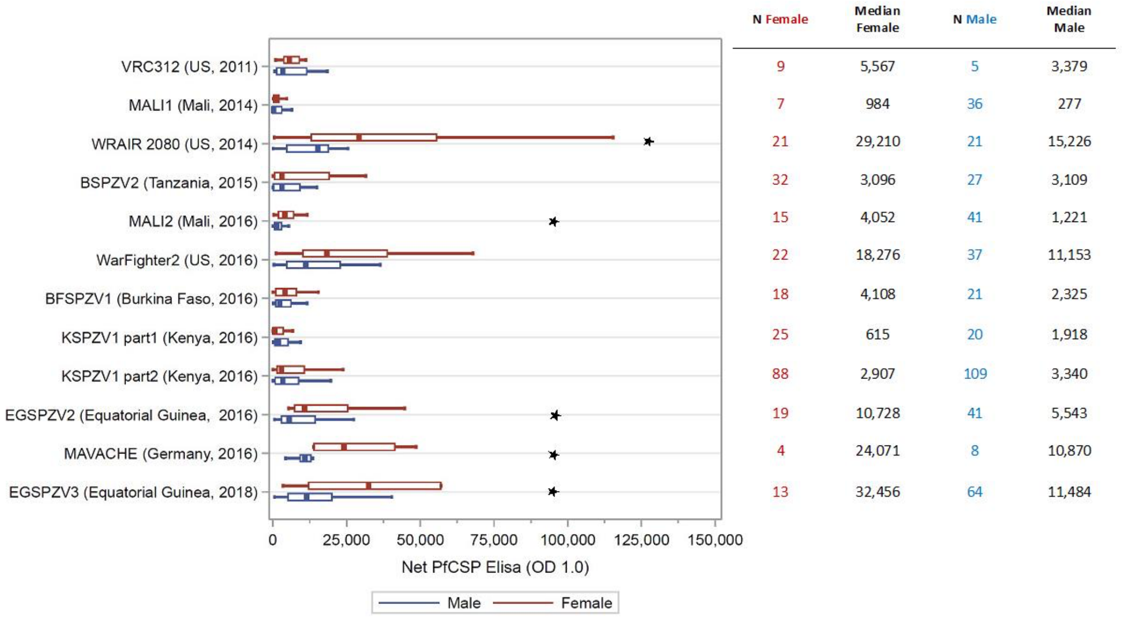

Figure S2. Net OD 1.0 PfCSP antibody levels by sex and vaccine efficacy (protected vs. not protected) for individual trials where significant differences were observed between males and females for antibody level (panels A-G). Each box plot includes median and interquartile ranges with the individual values represented (•). Results for males are in blue, females in red, with comparisons between protected participants by sex, not protected participants by sex and protected compared with not protected for each sex. In protected participants, net PfCSP antibody levels were higher in females in 6 of 7 trials with the difference significantly higher in 3 of 7 trials (WRAIR 2080 (US, 2014), MAVACHE (Germany, 2016) and EGSPZV3 (Equatorial Guinea, 2018)). Net OD 1.0 PfCSP antibody levels were also higher in 5 of 7 trials in female participants who were not protected.

A) VRC 312 (US, 2014)

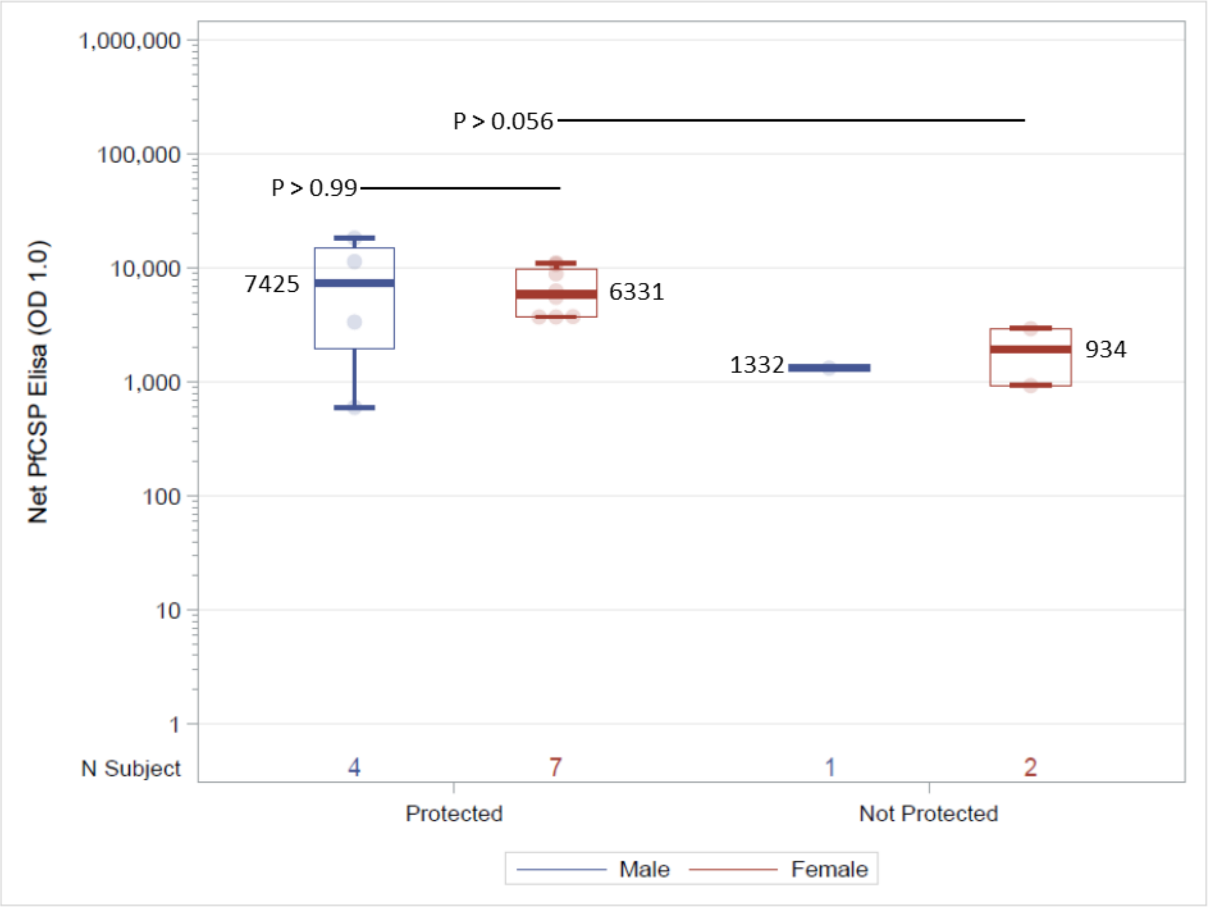

B) MLSZPV1 (Mali 1) (Mali, 2014)

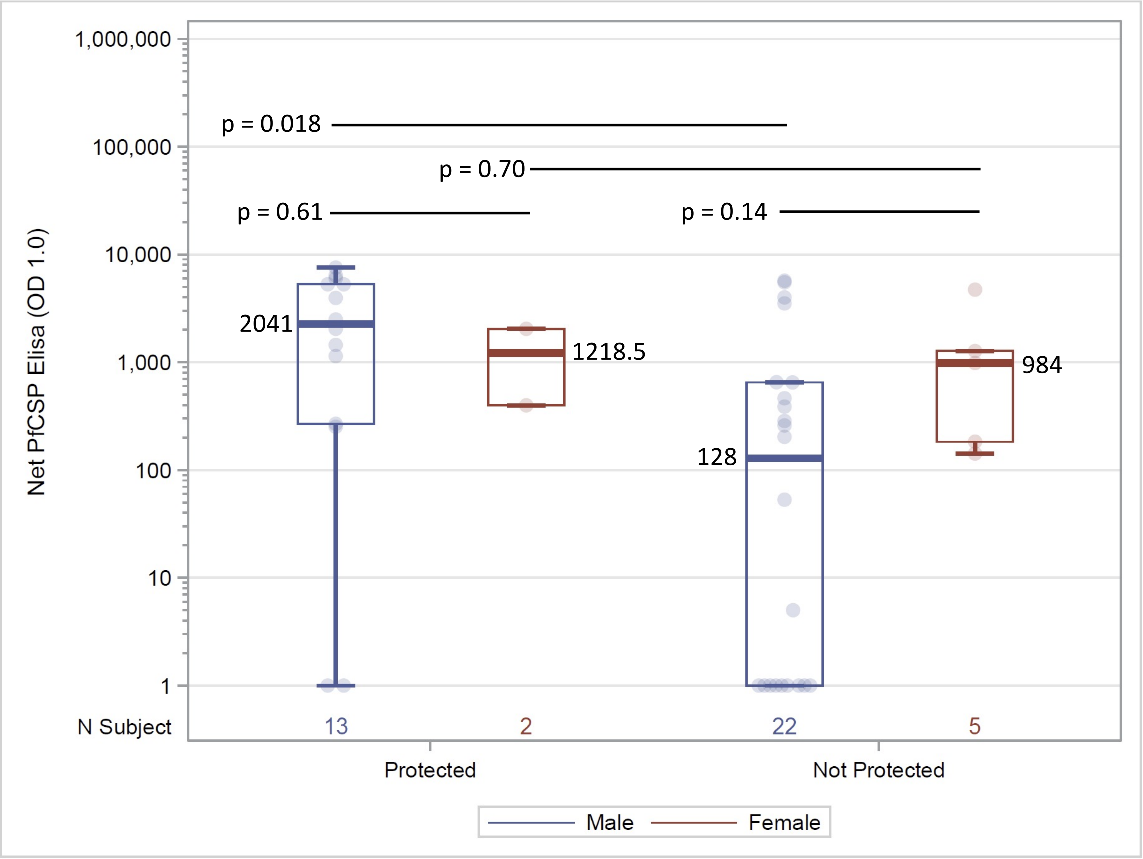

C) WRAIR 2080 (US, 2014)

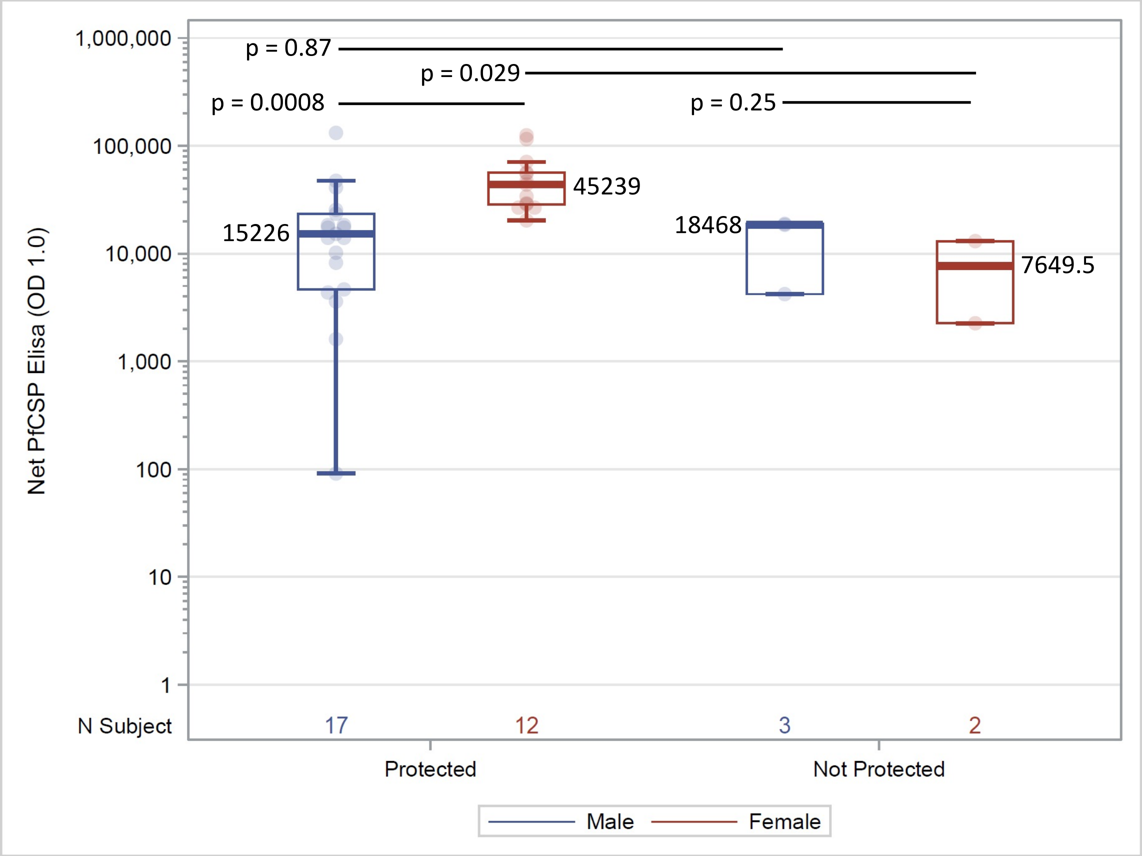

D) BSPZV2 (Tanzania, 2015)

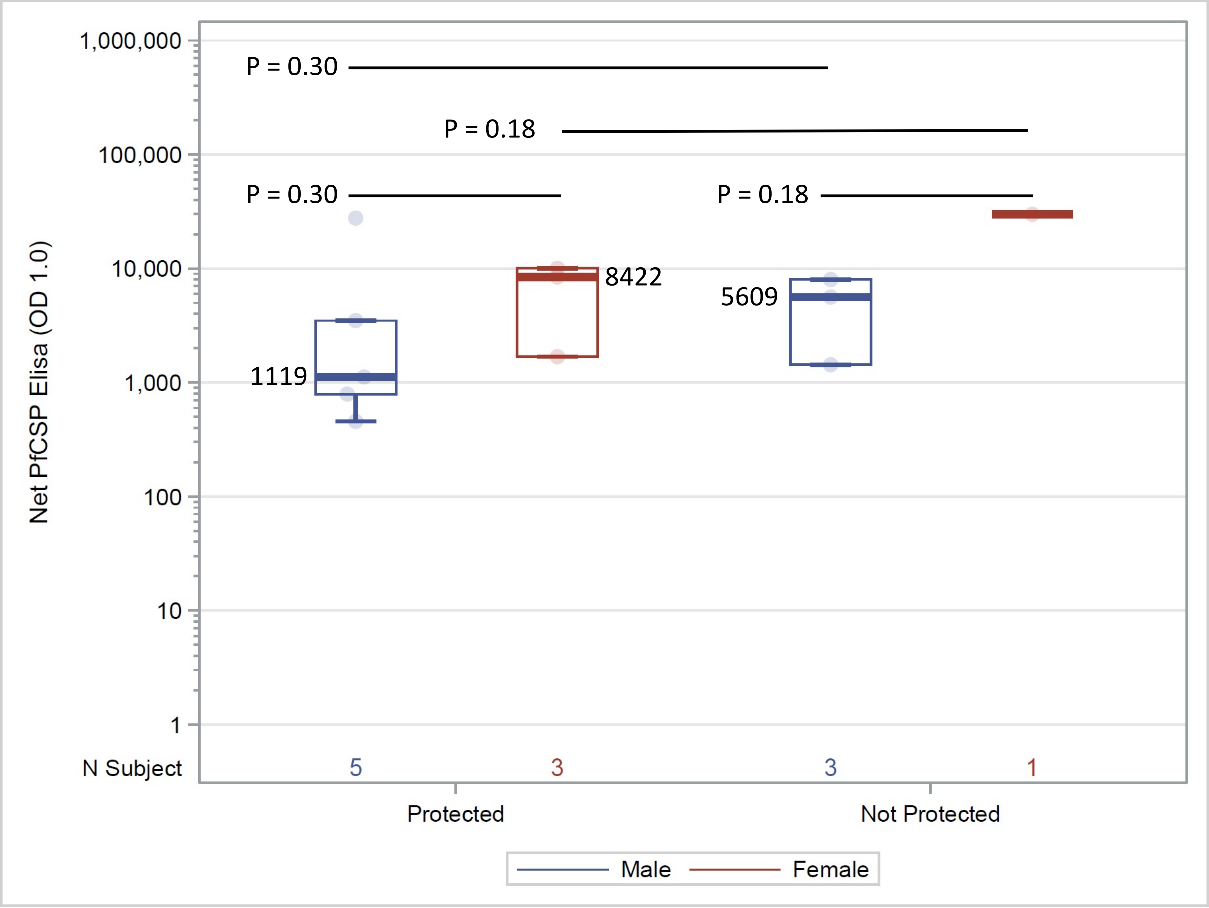

E) MLSPZV2 (Mali 2) (Mali, 2016)

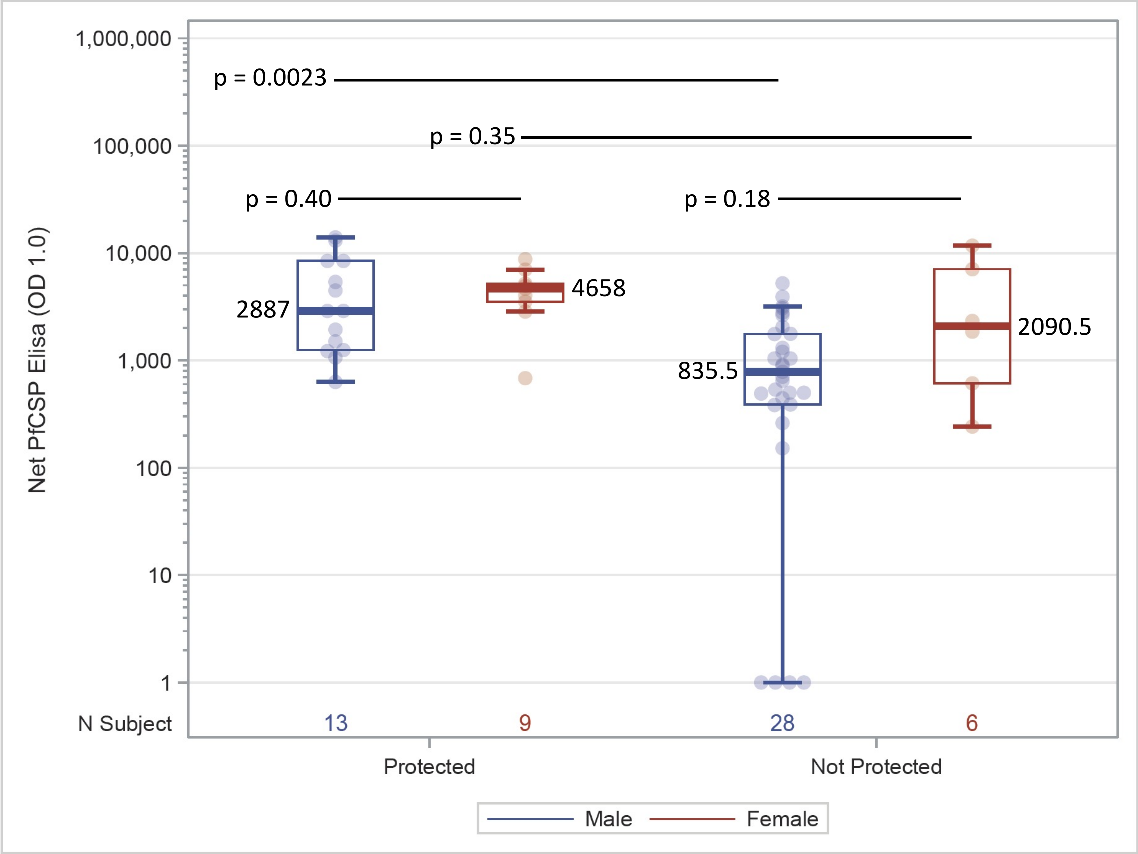

F) Warfighter 2 (US, 2016)

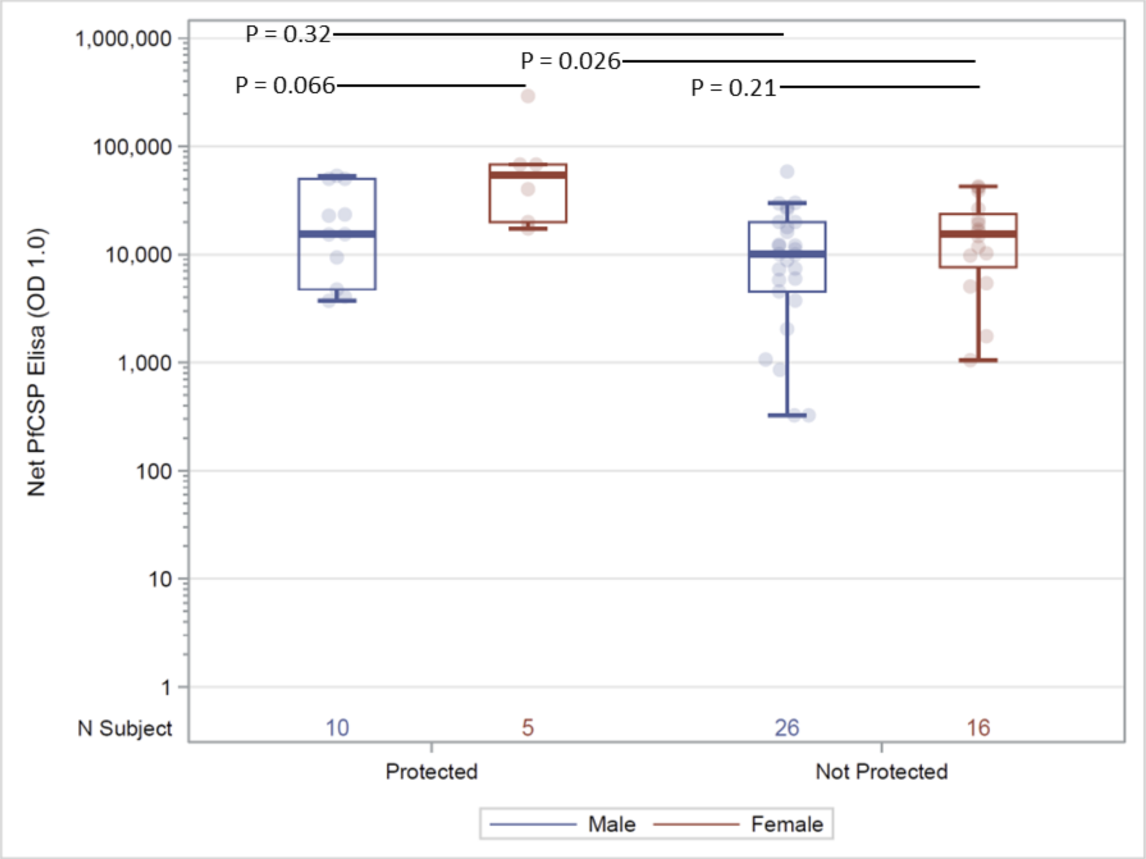

G) BFSPZV1 (Burkina Faso, 2016)

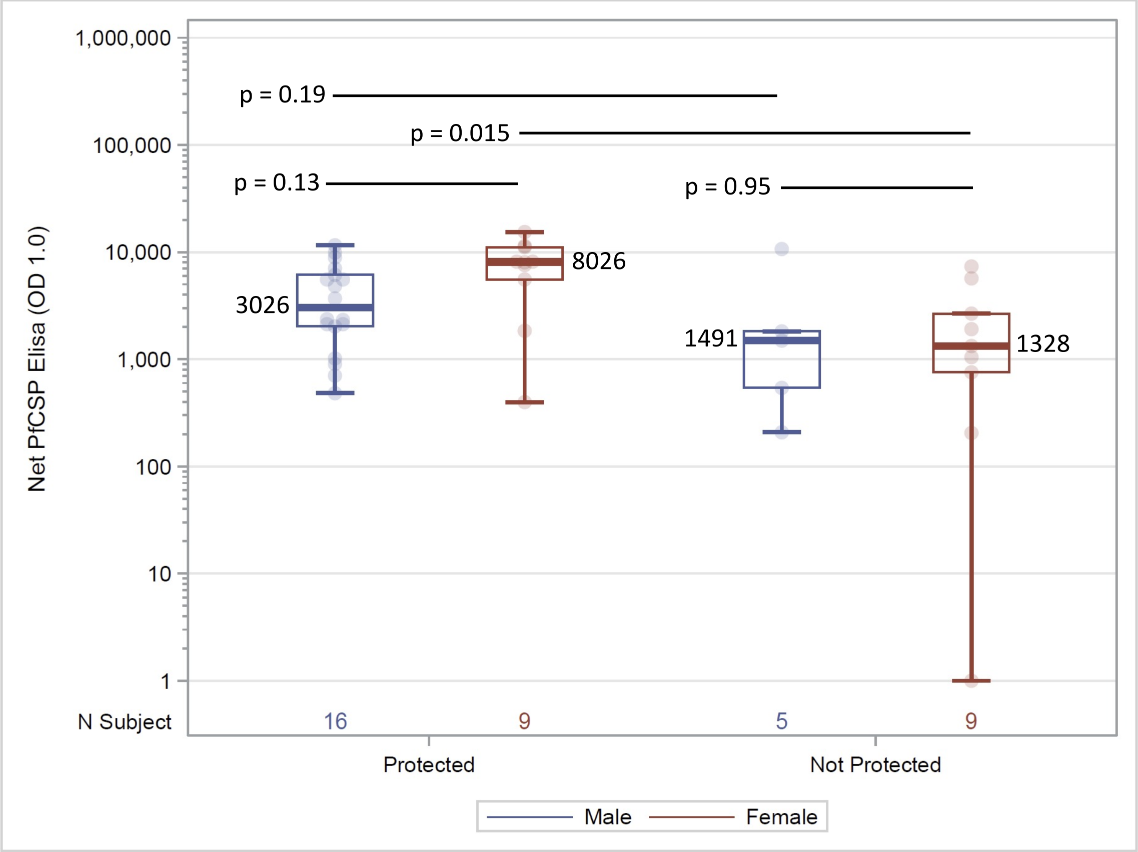

H) KSPZV1 Part 2 (Kenya, 2016)

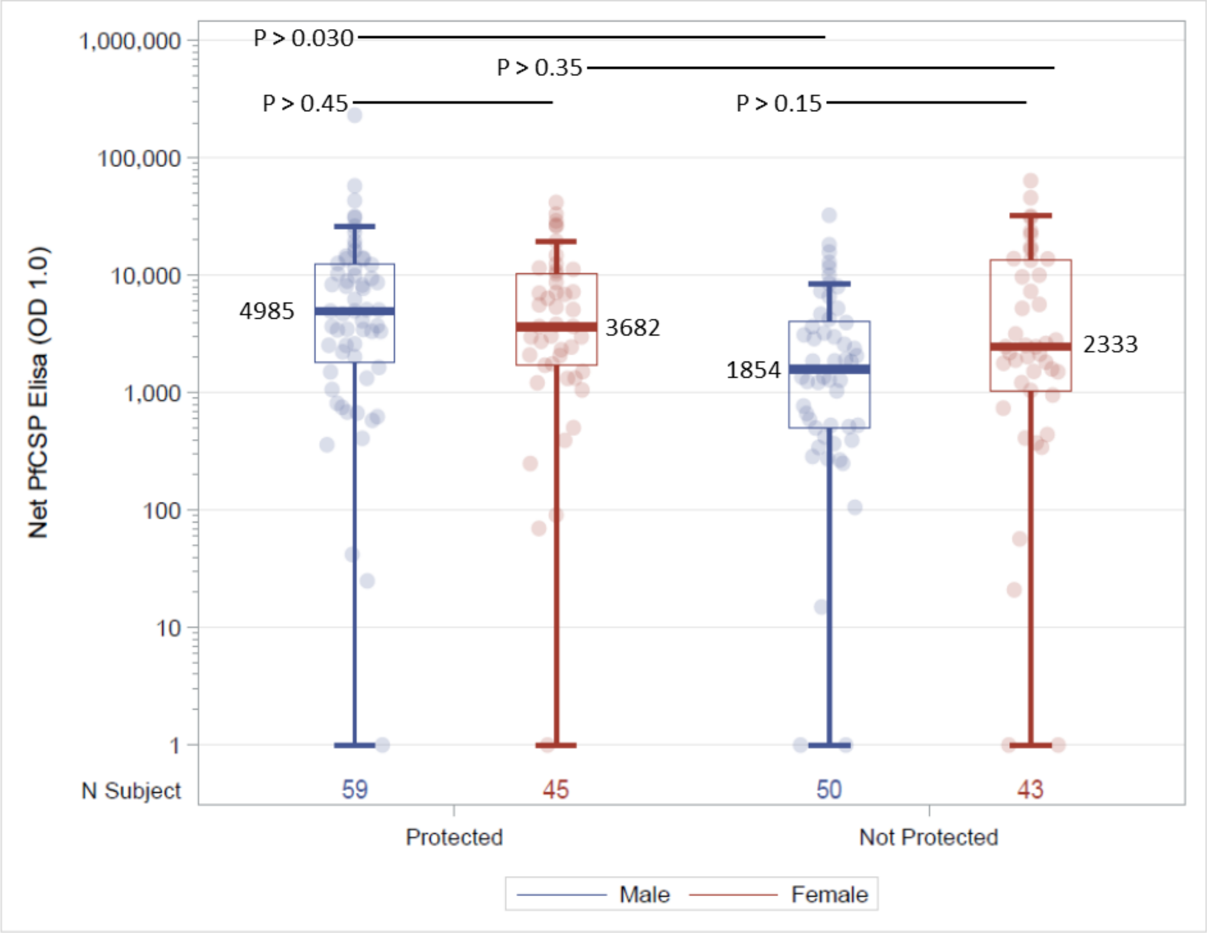

I) EGSPZV2 (Equatoria Guinea, 2016)

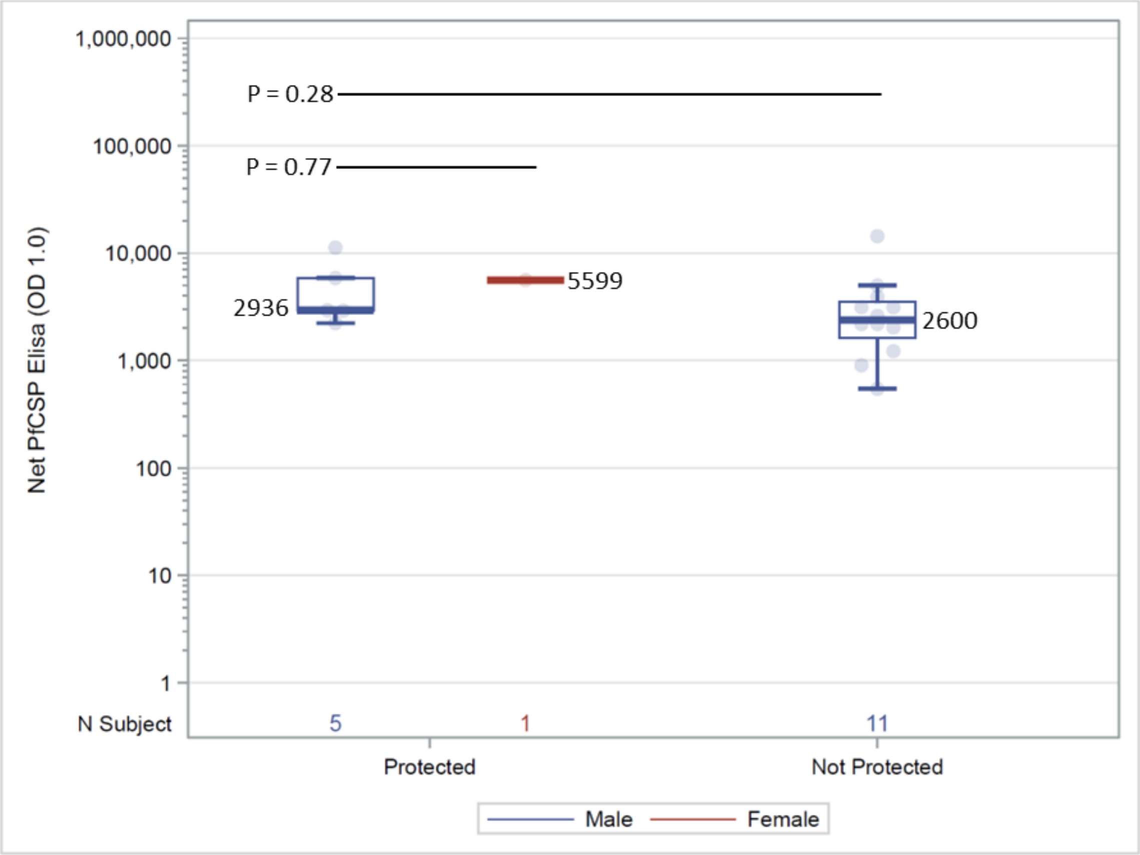

J) MAVACHE (Germany, 2016)

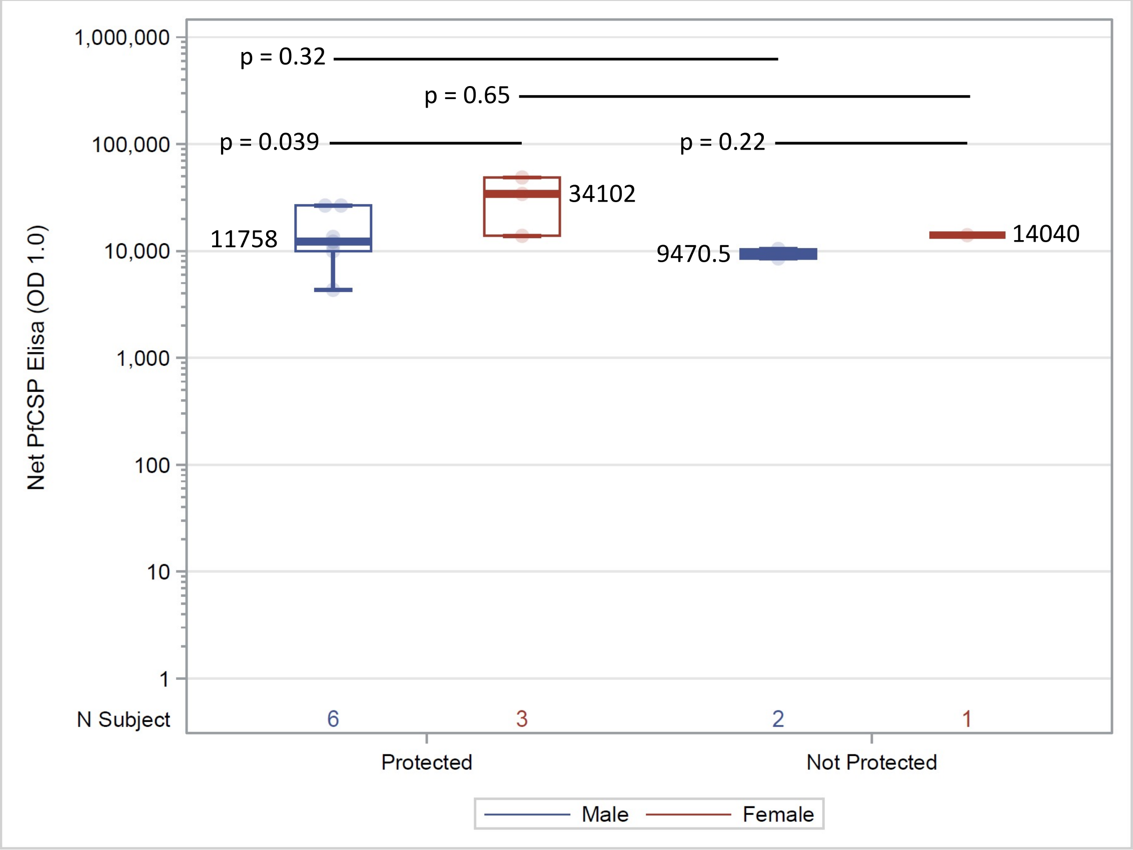

K) EGSPZV3 (Equatorial Guinea, 2018)

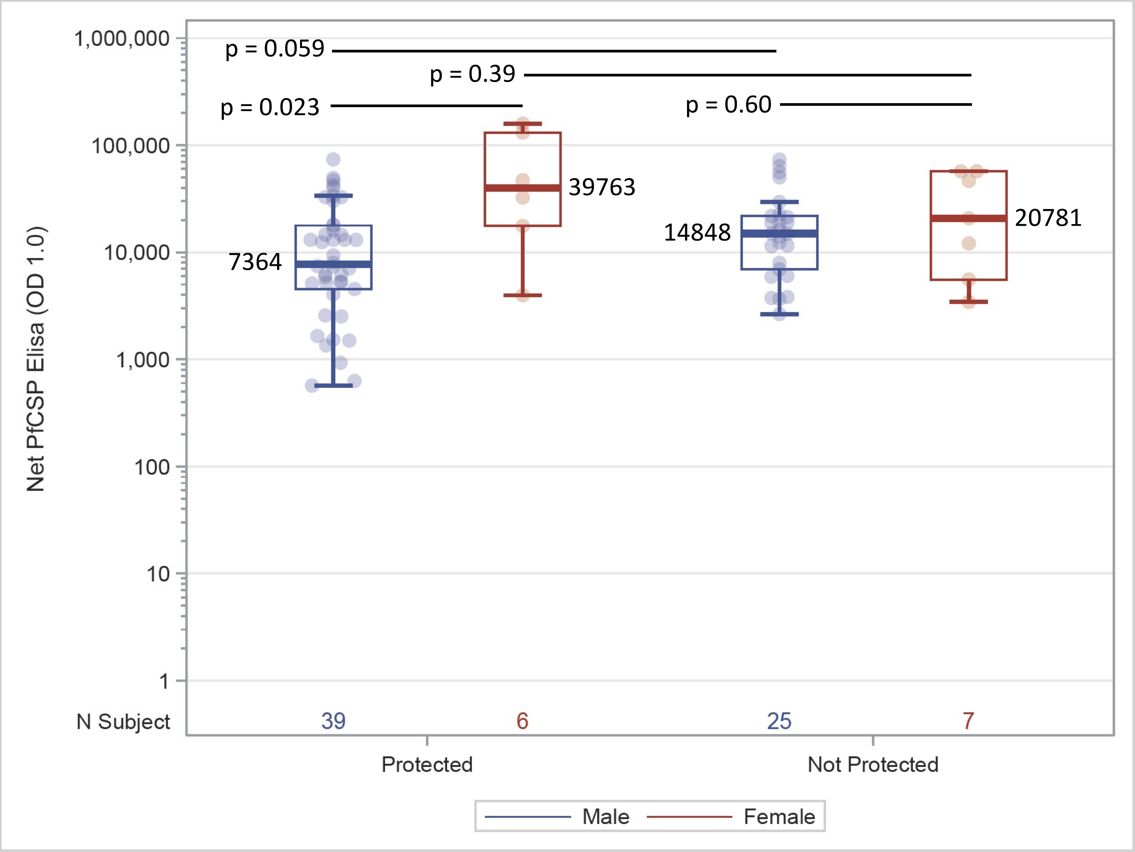

Detailed funding for each trial

VRC 312 (US, 2011)
The clinical trial was funded and supported by the National Institute of Allergy and Infectious Diseases (NIAID) Intramural Research Program. Production and characterization of the vaccine were supported in part by NIAID Small Business Innovation Research grants 4R44AI055229-08, 3R44AI055229-06S1, and 5R44AI058499-05. Production and characterization of the vaccine were supported in part by NIAID Small Business Innovation Research grants 4R44AI055229-08, 3R44AI055229-06S1, and 5R44AI058499-05

MLSZPV1 (Mali 1) (Mali, 2014) and MLSPZV2 (Mali 2) (Mali, 2016)
US National Institute of Allergy and Infectious Diseases, National Institutes of Health, Sanaria.

WRAIR 2080 (US, 2014)
Support was provided through the US Army Medical Research and Development Command, Military Infectious Diseases Research Program, and the Naval Medical Research Center's Advanced Medical Development Program

BSPZV2 (Tanzania, 2015)
This work supported by a public-private partnership, the Equatorial Guinea Malaria Vaccine Initiative (EGMVI), made up of the Government of Equatorial Guinea (EG) Ministries of Mines and Hydrocarbons, and Health and Social Welfare, Marathon EG Production Limited, Noble Energy, Atlantic Methanol Production Company, and EG Liquefied Natural Gas.

Warfighter 2 (US, 2016)
This work was supported by the Joint Warfighter Medical Research Program of the Department of Defense (W81XWH-15-C-0073; Warfighter 2; ClinicalTrials.gov number NCT02601716); Sanaria Inc., which supplied vaccine; the US Army Medical Research Acquisition Activity, with work performed by Sanaria with support from a US government contract (work unit number AI1507).

BFSPZV1 (Burkina Faso, 2016)
National Institutes of Health grant U01AI112367 (MBL)
National Institutes of Health grant 5R44AI055229 (SLH)

KSPZV1A; KSPZV1B (Kenya, 2016)
This work was supported by the National Institutes of Health Vaccine Research Center. Manufacturing and quality control release and stability assays for PfSPZ Vaccine were supported in part by National Institutes of Allergy and Infectious Diseases, National Institutes of Health SBIR grants 5R44AI055229- 09A1 and 2R44AI058375-06A1, awarded to SLH.

EGSPZV2 (Equatorial Guinea, 2016) and EGSPZV3 (Equatorial Guinea, 2018)

This work supported by a public-private partnership, the Equatorial Guinea Malaria Vaccine Initiative (EGMVI), made up of the Government of Equatorial Guinea (EG) Ministries of Mines and Hydrocarbons, and Health and Social Welfare, Marathon EG Production Limited, Noble Energy, Atlantic Methanol Production Company, and EG Liquefied Natural Gas.

MAVACHE (Germany, 2016)
The clinical trial was funded by the Deutsches Zentrum für Infektionsforschung (DZIF).

Manufacture of PfSPZ Vaccine, PfSPZ Challenge (NF54) and PfSPZ Challenge (7G8) was funded in part by the National Institute of Allergy and Infectious Diseases of the National Institutes of Health under SBIR award numbers 5R44AI058375 and 5R44AI055229. Both funders had no role in planning, conduct, analysis and publication of the results.
